# Higher urinary nitrate was associated with lower prevalence of congestive heart failure: results from NHANES

**DOI:** 10.1186/s12872-020-01790-w

**Published:** 2020-11-25

**Authors:** Zhuo Wu, Ting Tian, Wang Ma, Wen Gao, Ninghong Song

**Affiliations:** 1 The First Affiliated Hospital of Nanjing Medical University, Jiangsu Province Hospital, Nanjing, 210029 Jiangsu China; 2 Institute of Food Safety and Assessment, Jiangsu Provincial Center for Disease Control and Prevention, Nanjing, 210009 China

**Keywords:** Urinary nitrate, Cardiovascular diseases, Congestive heart failure, Logistic regression model, Complex sample, NHANES

## Abstract

**Background:**

Some studies have reported that nitrate intake from vegetables was inversely associated with many vascular diseases, but few studies have paid attention to the relationship between urinary nitrate and cardiovascular diseases (CVDs). This cross-sectional study aimed to explore the connections between urinary nitrate and prevalence of CVDs.

**Methods:**

The data of this study was collected from National Health and Nutrition Examination Survey (NHANES). Finally, several years’ data of NHANES were merged into 14,894 observations. Logistic regression models were used to examine the associations between urinary nitrate and CVDs by using the “survey” package in R software (version 3.2.3).

**Results:**

In the univariable logistic analysis, significant association was discovered between urinary nitrate and congestive heart failure, coronary heart disease, angina pectoris, myocardial infarction (all *P* < 0.001). By adjusting related covariates, the multivariable logistic analysis showed that the significant association only existed between urinary nitrate and congestive heart failure (OR = 0.651, 95% CI 0.507–0.838, *P* < 0.001). Compared to Q1 urinary nitrate level as reference, the risk for prevalent heart failure diminished along with increasing levels of urinary nitrates, (OR of Q2 level = 0.633, 95% CI 0.403–0.994), (OR of Q3 level = 0.425, 95% CI 0.230–0.783), (OR of Q4 level = 0.375, 95% CI 0.210–0.661), respectively. Moreover, urinary nitrate levels were associated with congestive heart failure in a dose-dependent manner in both 20–60 years group, 60+ years group and male, female group (*P* < 0.001, *P* = 0.011 and *P* = 0.009, *P* = 0.004).

**Conclusions:**

Independent of related covariates, higher urinary nitrate was associated with lower prevalent congestive heart failure.

## Background

In the past, nitrate was considered to be associated with detrimental health outcomes such as cancer [1, 2], however, understanding of the health impact of nitrate has recently undergone radical changes. In recent years, many researchers implemented studies on the relationship between dietary nitrate and vascular health [1–4]. It has been found that a short-term increase in nitrate intake can lower blood pressure [2] and improve endothelial function [4]. The circulation nitrate in human body mainly originate from two ways: oxidation of endogenously produced nitric oxide (NO) and our diet [5]. Dietary nitrate, abundant in beetroot and green leafy vegetables, is a potential and important alternative source for the production of NO via the nitrate–nitrite–NO pathway [6]. Nitrate from diet is rapidly absorbed in the small intestine and approximately 75% will ultimately be excreted in the urine [7, 8]. Thus, urinary nitrate can better reflect the level of nitrate in human body.

Cardiovascular diseases (CVDs) is the leading cause of death for entire population around the world [9]. Many studies have reported and demonstrated that increased dietary nitrate intake can decrease the risk of vascular diseases [10–13]. As is well known that endothelial dysfunction, especially impaired endothelium-dependent vasodilation mediated by NO, is a crucial early event in the development of CVDs [14]. However, up to now, few studies figured out the relationship between urinary nitrate and CVDs. Therefore, we tried to explore in the study.

## Methods

### Data source and study population

**Fig. 1:**
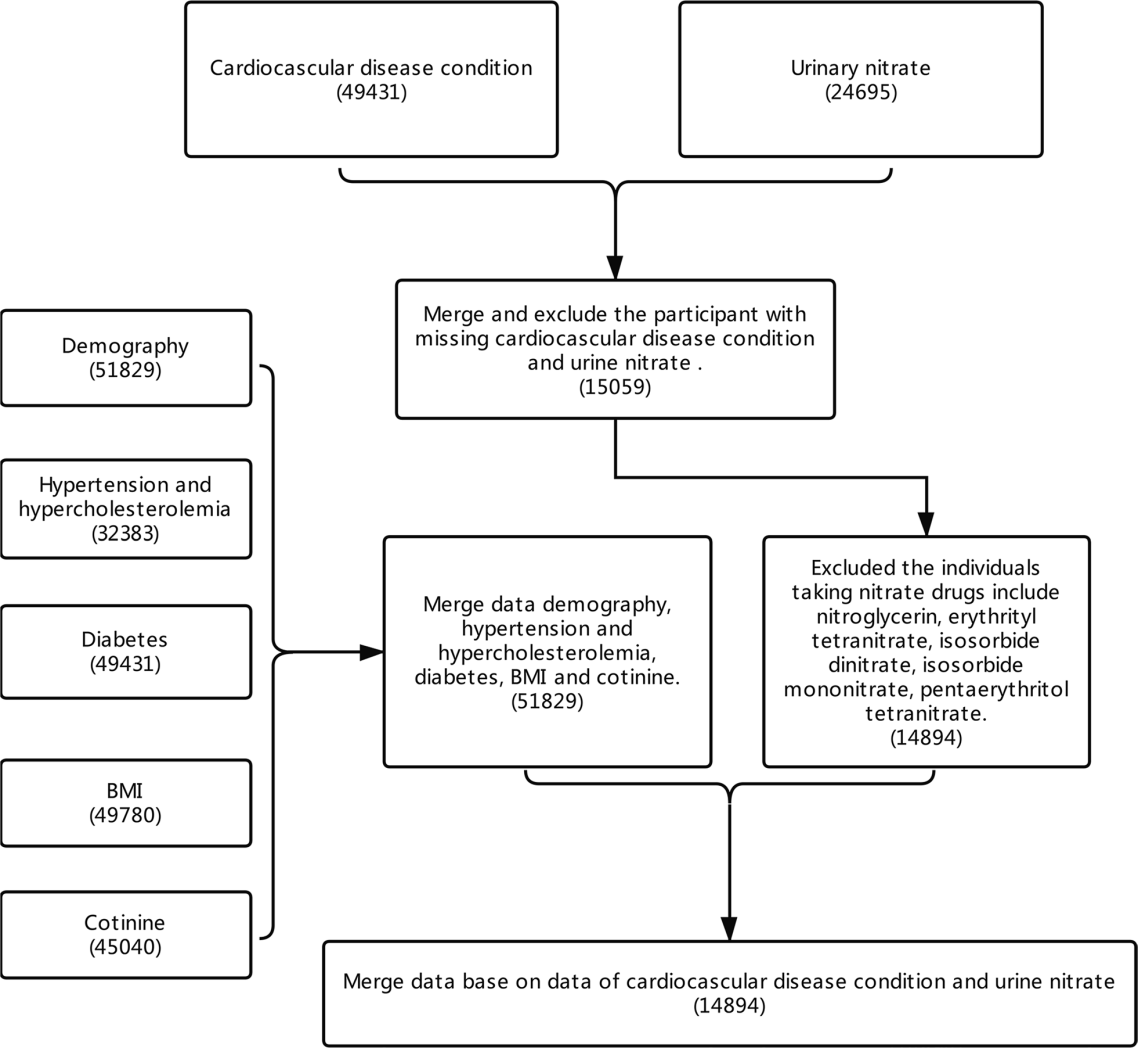
Participant flowchart

The National Health and Nutrition Examination Survey (NHANES) is an ongoing cross-sectional monitoring survey of the non-institutionalized US civilians, which were managed by the US National Center for Health Statistics (Centers for Disease Control and Prevention, Atlanta, GA, USA) [15]. It was conducted by complex survey design including oversampling, survey non-response, and post-stratification, thus selecting a representative US sample. The survey examines a nationally representative sample of about 5000 persons each year. These persons are located in counties across the country, 15 of which are visited each year. Sample weights were set uniquely for each participant for accounting the complex survey design in NHANES. Physical examinations, clinical laboratory examinations, and related measurement procedures were collected by Mobile Examination Center. This cross-sectional study data was collected from 5 surveys (2001–2002, 2005–2006, 2007–2008, 2009–2010 and 2011–2012). Besides, participants with missing information of CVDs and urinary nitrate were excluded in this study. Then demography, hypercholesterolemia, diabetes, hypertension, BMI and cotinine information of each subjects were extracted. All the data were finally merged into totally 14,894 observations. Details were shown on Fig. 1.

### Urinary nitrate measurement

Nitrate were measured in all available urine samples from 1/3 subsets of study participants aged over 6 years old in NHANES 2001–2002, 2009–2010 and 2011–2012. In 2005–2006 and 2007–2008 NHANES, nitrate was tested in participants aged over 6 years old. Urine specimens for urinary nitrate testing were processed, stored, and transferred to the Division of Environmental Health Laboratory Sciences, National Center for Environmental Health, and Centers for Disease Control and Prevention. All details for the urine specimen collection and processing were elucidated in the NHANES LPM (NHANES Laboratory/Medical Technologists Procedures Manual). All samples were stored at − 20 °C before being transported to National Center for Environmental Health for testing. Samples were analyzed for nitrate by using ion chromatography tandem mass spectrometry [16]. The NHANES kept to the quality assurance and quality control (QA/QC) protocols which met the 1988 Clinical Laboratory Improvement Act mandates. Details can been in the NHANES LPM.

### Cardiovascular diseases

In NHANES, the CVDs were provided by a self-reported personal interview. Among the tested participants who needed to answer the CVDs conditions were all over 20 years old. Participants would be considered to have CVDs including congestive heart failure, coronary heart disease, angina and myocardial infarction if they confirmed yes to the questions “Has a doctor or other health professional ever told you that you had congestive heart failure?”, “Has a doctor or other health professional ever told you that you had coronary heart disease?”, “Has a doctor or other health professional ever told you that you had angina, also called angina pectoris?”, “Has a doctor or other health professional ever told you that you had a heart attack (also called myocardial infarction)?” respectively. These questions were included in the medical conditions part of the interview survey, and the diseases condition of participants were based on the medical history diagnosed by the clinicians or medical institutions. Besides, the explanations in the manuals described these diseases condition and differences which can make it easier for the interviewer and participants to understand, and we displayed them as followings.

*Congestive heart failure* Is when the heart can't pump enough blood to the body. Blood and fluid “back up” into the lungs, which makes you short of breath. Heart failure causes fluid buildup in and swelling of the feet, legs and ankles.

#### Interviewer

Do not count heart murmurs, irregular heart beats, chest pain or heart attacks.

*Coronary heart disease* Is when the blood vessels that bring blood to the heart muscle become narrow and hardened due to plaque (plak). Plaque buildup is called atherosclerosis (ATH-er-o-skler-O-sis). Blocked blood vessels to the heart can cause chest pain or a heart attack.

#### Interviewer

If the respondent reports chest pain, probe if a doctor told them that they had blocked blood vessels or coronary heart disease.

*Angina (Angina Pectoris)* (AN-ji-na or an-JI-na). Angina is chest pain or discomfort that occurs when the heart does not get enough blood.

#### Interviewer

If the respondent reports chest pain, probe if a doctor told them that they had blocked blood vessels or angina.

*Heart attack (Myocardial Infarction)* A heart attack happens when there is narrowing of a blood vessel that supplies the heart. A blood clot can form and suddenly cut off the blood supply to the heart muscle. This damage causes crushing chest pain that may also be felt in the arms or neck. There can also be nausea, sweating, or shortness of breath.

### Covariates

A wide range of socio-demographic variables and medical histories were collected in NHANES including sex, age (years), ethnicity (Mexican American, Other Hispanic, Non-Hispanic White, Non-Hispanic Black and Other Race—Including Multi-Racial), BMI (Individuals were divided as overweight if their BMI value ranging from 25 to 30 kg/m^2^, and obese if their BMI ≥ 30 kg/m^2^ on the basis of the World Health Organization (WHO) standards), serum cotinine (non-smoker defined as serum cotinine ≤ 10 ng/mL and smoker defined as serum cotinine > 10 ng/mL) [17], poverty income ratio (PIR) [poverty: PIR ≤ 1, beyond the poverty threshold: PIR > 1], hypertension, hypercholesterolemia and diabetes history. The history of hypertension, hypercholesterolemia and diabetes were defined as participants confirmed yes to the questions “Have you ever been told by a doctor or other health professional that you had hypertension, also called high blood pressure?”, “Have you ever been told by a doctor or other health professional that your blood cholesterol level was high?” and “Have you ever been told by a doctor or health professional that you have diabetes or sugar diabetes?”.

### Statistical analyses

All statistical analysis was on the R software (version 3.2.3). Logistic regression models with were used to examine the associations between urinary nitrate and CVDs by using the “survey” package. Each analysis used sample weights to estimate representatives of the US population. Logistic regression models with complex sample were applied to test the associations between CVDs and urinary nitrate adjusted by covariates (age, sex, ethnicity, serum cotinine, BMI, PIR, hypertension, hypercholesterolemia and diabetes history). We would perform logistic analysis twice, taking urinary nitrate as continuous variables and categorical factors respectively. In the model which used continuous urinary nitrate, urinary nitrate was normalized for creatinine as follows: *urinary nitrate concentration (ng/mL)/(urinary creatinine (mg/dL)/100)* = *μg nitrate/g creatinine*. Then normalized nitrate was transferred by the natural log transformation for the significant deviation from the normal distribution. In the model using categorical urinary nitrate, the level of urinary nitrates was divided into quartiles (Q1: ≤ 26,100; Q2: 26,100–45,000; Q3: 45,000–70,600; Q4: > 70,600) by the interquartile. Both models were applied for the stratified analysis by genders and age groups (younger people age 20–60 years and older people age over 65 years) between CVDs and nitrate. All significance tests were two-sided with significant threshold of 0.05.

## Results

### General demographic information

14,894 participants were included in this study from 5 NHANES surveys. Demographic characteristics of all participants and different urinary nitrate groups were shown in Table 1. Genders had a balance of distribution which female accounted for 51.36%. The prevalence of hypertension, hypercholesterolemia and diabetes were 32.86% (4883), 41.21% (4415) and 11.10% (1626) respectively. Participants with any of the congestive heart failure, coronary heart disease, angina and myocardial infarction had lower urinary nitrate levels, demonstrated in Additional file 1: Table S1.

**Table 1:** Baseline characteristics of all study participants divided by urinary nitrate groups

Variables	All participants, n = 14,894	Urinary nitrate (ng/mL)^a^				*P*
		Q1, n = 3714	Q2, n = 3736	Q3, n = 3708	Q4, n = 3736	
Age	48.67 (18.00)	53.29 (18.48)	50.09 (18.10)	47.11 (17.66)	44.22 (16.44)	< 0.001
Gender						< 0.001
Male (N, %)	7245 (48.64)	1415 (38.10)	1761 (47.14)	2002 (53.99)	2067 (55.33)	
Female (N, %)	7649 (51.36)	2299 (61.90)	1975 (52.86)	1706 (46.01)	1669 (44.67)	
Race (N, %)						< 0.001
Mexican American	2668 (17.91)	614 (16.53)	614 (18.09)	686 (18.50)	692 (18.52)	
Other Hispanic	1175 (7.89)	286 (7.70)	293 (7.84)	306 (8.25)	290 (7.76)	
Non-Hispanic White	7011 (47.07)	1916 (51.59)	1740 (46.57)	1653 (44.58)	1702 (45.56)	
Non-Hispanic Black	3184 (21.38)	710 (19.22)	846 (22.64)	862 (23.25)	766 20.50)	
Other race—including multi-racial	856 (5.75)	188 (5.06)	181 (4.84)	201 (5.42)	286 (7.66)	
PIR (N, %)						0.276
≤ 1	2719 (19.74)	660 (19.32)	657 (19.02)	671 (19.51)	731 (21.12)	
> 1	11,052 (80.26)	2757 (80.68)	2797 (80.98)	2786 (80.49)	2730 (78.88)	
Cotinine (N, %)						< 0.001
≤ 0.01						
0.01–10	10,547 (74.44)	2900 (82.18)	2749 (77.22)	2585 (73.31)	2313 (65.10)	
> 10	3621 (25.56)	629 (17.82)	811 (22.78)	941 (26.69)	1240 (34.90)	
BMI (N, %)						< 0.001
< 25	4448 (30.33)	1231 (33.91)	1118 (30.31)	979 (26.81)	1120 (30.33)	
25–30	5019 (34.23)	1228 (33.83)	1268 (34.37)	1289 (35.30)	1234 (33.41)	
> 30	5197 (35.44)	1171 (32.26)	1303 (35.32)	1384 (37.90)	1339 (36.26)	
Hypertension (N, %)						< 0.001
No	9977 (67.14)	2208 (59.53)	2397 (64.31)	2595 (70.23)	2777 (74.47)	
Yes	4883 (32.86)	1501 (40.47)	1330 (35.69)	1100 (29.77)	952 (25.53)	
Hypercholesterolemia (N, %)						< 0.001
No	6298 (58.79)	1618 (56.75)	1579 (56.92)	1569 (60.30)	1532 (61.63)	
Yes	4415 (41.21)	1233 (43.25)	1195 (43.08)	1033 (39.70)	954 (38.37)	
Diabetes (N, %)						< 0.001
No	13,017 (88.90)	3118 (85.31)	3229 (87.91)	3265 (89.80)	3405 (92.55)	
Yes	1626 (11.10)	537 (14.69)	444 (12.09)	371 (10.20)	274 (7.45)	

*PIR* poverty impact ratio, *BMI* body mass index, *Cotinine* the predominate metabolite of nicotine

^a^Urine nitrate was divided to four levels by quartile (Q1: ≤ 26,100; Q2: 26,100–45,000; Q3: 45,000–70,600; Q4: > 70,600)

### Association between urinary nitrate and CVDs

To explore whether urinary nitrate levels were associated with any CVDs, logistic regression methods were used by conducting univariable and multivariable analyses. Pronounced association were found between urinary nitrate levels and prevalent congestive heart failure, coronary heart disease, angina pectoris and myocardial infarction in the univariable logistic analysis (all *P* < 0.001) (Tables 2, 3). After adjusting related covariates, the multivariable logistic analysis showed that the significant association only existed between urinary nitrate and occurrence of congestive heart failure (OR = 0.651, 95% CI 0.507–0.838, *P* < 0.001), details were displayed in Table 2. Furthermore, the concentration of urinary nitrate was divided into quartiles (Q1, Q2, Q3 and Q4) according to the interquartile. Compared to Q1 which was reference group, the Q2, Q3 and Q4 had pronounced negative correlation with congestive heart failure (OR = 0.633, 95% CI 0.403, 0.994), (OR = 0.425, 95% CI 0.230, 0.783), (OR = 0.375, 95% CI 0.210, 0.661), respectively. Trend test showed statistical significance (*P* < 0.001) in Table 3.

**Table 2:** Logistic regression results for relationship between urinary nitrate (log transformation) and various cardiovascular diseases

	OR (95%CI)^a^	*P*	OR (95%CI)^b^	*P*
Congestive heart failure	0.980 (0.976, 0.984)	< 0.001	0.651 (0.507, 0.838)	< 0.001
Coronary heart disease	0.989 (0.985, 0.993)	< 0.001	1.052 (0.776, 1.426)	0.745
Angina pectoris	0.992 (0.988, 0.996)	< 0.001	0.894 (0.720, 1.110)	0.309
Myocardial infarction	0.990 (0.985, 0.994)	< 0.001	1.076 (0.869, 1.331)	0.503

^a^Model was not adjusted by any covariate

^b^Model was adjusted by age, gender, race/ethnicity, poverty income ratio, cotinine, BMI, hypertension, hypercholesterolemia and diabetes (poverty income ratio: Q1: ≤ 1, Q2: > 1; cotinine: Q1: ≤ 0.01, Q2: 0.01–10, Q3: > 10; BMI: Q1: < 25; Q2: 25–30; Q3: > 30)

**Table 3:** Logistic regression results for relationship between urinary nitrate levels and various cardiovascular diseases

	OR (95% CI)^a^	*P* for trend	OR (95% CI)^b^	*P* for trend
Congestive heart failure				
Q1	Ref	< 0.001	Ref	< 0.001
Q2	0.978(0.971, 0.986)		0.633 (0.403, 0.994)	
Q3	0.970 (0.964, 0.977)		0.425 (0.230, 0.783)	
Q4	0.967 (0.960, 0.973)		0.375 (0.210, 0.661)	
Coronary heart disease				
Q1	Ref	< 0.001	Ref	0.567
Q2	1.000 (0.992, 1.008)		0.981 (0.618, 1.555)	
Q3	0.986 (0.978, 0.993)		0.632 (0.365, 1.094)	
Q4	0.983 (0.975, 0.991)		1.003 (0.586, 1.717)	
Angina pectoris				
Q1	Ref	< 0.001	Ref	0.745
Q2	0.994 (0.988, 1.001)		0.800 (0.446, 1.437)	
Q3	0.990 (0.984, 0.996)		0.462 (0.252, 0.846)	
Q4	0.989 (0.982, 0.995)		0.697 (0.372, 1.303)	
Myocardial infarction				
Q1	Ref	< 0.001	Ref	0.617
Q2	0.992 (0.984, 0.983)		0.785 (0.499, 1.233)	
Q3	0.984 (0.976, 0.992)		0.751 (0.437, 1.288)	
Q4	0.983 (0.975, 0.991)		0.913 (0.535, 1.558)	

^a^Model was not adjusted by any covariate

^b^Model was adjusted by age, gender, race/ethnicity, poverty income ratio, cotinine, BMI, hypertension, hypercholesterolemia and diabetes. (poverty income ratio: Q1: ≤ 1, Q2: > 1; cotinine: Q1: ≤ 0.01, Q2: 0.01–10, Q3: > 10; BMI: Q1: < 25; Q2: 25–30; Q3: > 30)

### Stratification analysis

In view of the different distribution of congestive heart failure in various age and gender groups, further stratification logistic analyses were performed. Corresponding results were listed in Table 4. In the 20–60 years group, urinary nitrate levels were associated with congestive heart failure in a dose-dependent manner (*P* < 0.001). Compared with Q1 reference group, risk of having congestive heart failure in the Q3 group (OR = 0.224, 95% CI 0.082, 0.608) and Q4 group (OR = 0.195, 95% CI 0.071, 0.534) were significantly lower. It was found that urinary nitrate levels had significantly negative correlation with congestive heart failure in > 60 years group (*P* = 0.011). Q4 group had lower congestive heart failure than Q1 group (OR = 0.485, 95% CI 0.251, 0.936). In addition, regardless of genders, the inverse relationship between urinary nitrate and congestive heart failure still existed in both male and female groups (*P* = 0.009, *P* = 0.004, respectively). There were no connections between urinary nitrate and coronary heart disease, angina in each gender and age groups. Furthermore, we performed age stratification in each sex group, as we can see from Table 5, the significant association existed in every subgroup except in > 60 years female subgroup.

**Table 4:** Logistic regression results for relationship between urinary nitrate levels and congestive heart failure stratified by age groups and genders respectively

Urinary nitrate^a^	20–60 year OR (95%CI)	60+ year OR (95%CI)	Male OR (95%CI)	Female OR (95%CI)
Q1	Ref	Ref	Ref	Ref
Q2	0.621 (0.287, 1.343)	0.621 (0.359, 1.077)	0.629 (0.343, 1.152)	0.690 (0.354, 1.344)
Q3	0.224 (0.082, 0.608)	0.508 (0.255, 1.011)	0.356 (0.138, 0.915)	0.600 (0.290, 1.242)
Q4	0.195 (0.071, 0.534)	0.485 (0.251, 0.936)	0.441 (0.217, 0.899)	0.201 (0.076, 0.530)
*P* for trend	< 0.001	0.011	0.009	0.004

All models were adjusted by age, gender, race/ethnicity, poverty income ratio, cotinine, BMI, hypertension, hypercholesterolemia and diabetes (poverty income ratio: Q1: ≤ 1, Q2: > 1; cotinine: Q1: ≤ 0.01, Q2: 0.01–10, Q3: > 10; BMI: Q1: < 25; Q2: 25–30; Q3: > 30)

^a^Urinary nitrate was divided to four levels by quartile (Q1: ≤ 26,100; Q2: 26,100–45,000; Q3: 45,000–70,600; Q4: > 70,600)

**Table 5:** Logistic regression results for relationship between urinary nitrate levels and congestive heart failure stratified by age groups and genders

	Male		Female	
Urinary nitrate^a^	20–60	60+	20–60	60+
Q1	Ref	Ref	Ref	Ref
Q2	0.829 (0.317, 2.166)	0.531 (0.249, 1.135)	0.344 (0.099, 1.186)	0.807 (0.376, 1.703)
Q3	0.138 (0.033, 0.582)	0.407 (0.160, 1.033)	0.507 (0.118, 2.171)	0.672 (0.287, 1.573)
Q4	0.262 (0.074, 0.925)	0.494 (0.214, 1.140)	0.058 (0.006, 0.519)	0.291 (0.099, 0.858)
*P* for trend	0.008	0.049	0.024	0.053

All models were adjusted by age, gender, race/ethnicity, poverty income ratio, cotinine, BMI, hypertension, hypercholesterolemia and diabetes (poverty income ratio: Q1: ≤ 1, Q2: > 1; cotinine: Q1: ≤ 0.01, Q2: 0.01–10, Q3: > 10; BMI: Q1: < 25; Q2: 25–30; Q3: > 30)

^a^Urinary nitrate was divided to four levels by quartile (Q1: ≤ 26,100; Q2: 26,100–45,000; Q3: 45,000–70,600; Q4: > 70,600)

## Discussion

Higher urinary nitrate was associated with lower prevalence of congestive heart failure from this cross-sectional study. Subjects with congestive heart failure had significant lower urinary nitrate levels. Nitrate and nitrite, previously known as unnecessary residues in the food chain, have potentially carcinogenic effects, especially positively related to gastric cancer [18]. Nowadays, it is widely recognized that nitrite and nitrate are important reservoirs of NO and crucial for maintenance of NO physiological levels [5]. Dietary nitrate can affect NO in the circulation in a dose-dependent manner by increasing the levels of nitrite, NO, and related nitroso compounds [19–22].Through the classic L-arginine-NO-synthase pathway, inert nitrogen anion circulates in vivo to form NO [23].

After adjusting the involved covariates, the negative correlation between CVDs and urinary nitrate only existed in patients with congestive heart failure. Worldwide, heart failure is a major public health problem with a total number more than 23 million and still rising [24]. Heart failure is the terminal conditions of many heart diseases, for instance, ischemic heart disease, coronary artery disease and atrial fibrillation. This disease has tremendous undesirable consequences which can lower the quality of life and even lead to death [25]. Numerous human intervention studies have investigated the effects of dietary nitrate on vascular health [3, 13, 26, 27]. These studies have shown that increased nitrate intake can improve vasorelaxation [27], lower blood pressure [2] and improve function of endothelium [4], which eventually resulted in lower death risk of atherosclerotic vascular disease (ASVD) [10]. NO is an endothelium-derived relaxing factor (EDRF) endogenously biosynthesized from L-arginine, oxygen, and Nicotinamide Adenine Dinucleotide Phosphate (NADPH) via various nitric oxide synthase (NOS) enzymes [28]. The vascular endothelium (inner lining) uses NO to give a signal of making surrounding smooth muscle relaxing, thus resulting in the process of vasodilation and increasing blood flow [29, 30]. In an intervention study, patients with systolic heart failure were well-tolerated for acute dietary NO_3_^−^ intake, and had enhanced NO bioavailability and exercise capacity [31]. In addition, NO_3_^−^ increased vasodilatory ability, cardiac output reserves and reduced arterial wave reflections in heart failure patients with Preserved Ejection Fraction [32].

About 65–70% of nitrate is excreted in urine in the 24 h and less than 1% is excreted into feces [33]. Urinary nitrate levels are generally considered as appropriate biomarkers for assessing chronic exposure. Nitrate in blood is also a good marker of human exposure; however, it is more susceptible to recent exposures. Therefore, urinary nitrate can represent stable and real nitrate concentration in human body [34–36]. At present, there are lots of researches at home and abroad focusing on the relationship between urinary nitrate and other diseases. For example, the decrease in urinary nitrate concentration in neonates with persistent pulmonary hypertension (PPHN) implied a role of NO in the pathogenesis of PPHN [37]. Furthermore, from the 2005–2006 National Health and Nutrition Examination Survey, Ko et al. [38] found the inverse association between urinary nitrate and thiocyanate concentrations in U.S. Adults.

Our data suggested that the remarkable relationships was existed in different gender and age groups. From self-reported data obtained from 2003 to 2006 National Health and Nutrition Examination Survey, the prevalence of heart failure increased with age, and males were higher than females. There are differences in gene expression and physiology between men and women, so that gender has a broad impact on human biology and disease development [39]. Studies have indicated that estrogen plays a protective role in the maintenance of cardiovascular health among females, reducing the occurrence of CVDs, thereby reducing the CVDs mortality rate in women. In our study, the association between urinary nitrate levels and congestive heart failure was no longer meaningful in > 60 year female subgroup. This could be explained by that the decrease of estrogen secretion in postmenopausal women [40].

Other studies had evaluated the relationship between nitrate and atherosclerotic vascular disease (ASVD), carotid atherosclerosis and ischemic cerebrovascular disease [10]. However, it is worth mentioning that our study comprehensively and systematically explored the association of urinary nitrate with various kinds of CVDs based on large samples from well-designed database. There are still some limitations in this study. For instance, exposure misclassification would exist because of imperfect single measurements of urinary nitrate as markers of chronic exposure. In addition, the cross-sectional study might hinder the real interpretation of causality from the results. The longitudinal study design is in necessity to understand the true association between nitrate and congestive heart failure. Besides, the definitions of these CVDs are based on the self-reported information in the face-to-face interview, there may be some bias which affected the results to some extent. However, previous scholars also do researches on the CVDs and other diseases based on the self-reported information from the NAHENS [41, 42]. The study design of NAHENS is reasonable, and investigators have received good training. When conducting the interview, a computer-aided investigation system can help participants easily understanding health-related questions. For the cardiovascular disease outcomes in this study, there are detailed problem description and explanation for each question. Based on the conclusion of medical diagnosis recall, the results are relatively reliable.

## Conclusions

Higher urinary nitrate was associated with lower prevalence of congestive heart failure and these significant results were still existed in most gender and age groups. This can remind us that raised intake of dietary nitrate may help resist the occurrence of congestive heart failure in particular populations.

## Supplementary information

**Additional file 1: Table S1**. The urinary nitrate level of various cardiovascular diseases.

## Data Availability

The NHANES database is available at: https://www.cdc.gov/nchs/nhanes/about_nhanes.htm. The datasets used and/or analyzed during the current study are available from the corresponding author on reasonable request.

## Abbreviations

CVD: Cardiovascular disease
CI: Confidence interval
OR: Odds ratio
NHANES: National Health and Nutrition Examination Surveys
NO: Nitric oxide
BMI: Body mass index
WHO: World Health Organization
PIR: Poverty income ratio
ASVD: Atherosclerotic vascular disease
