# Supplementary material for: Higher urinary nitrate was associated with lower prevalence of congestive heart failure: results from NHANES
Source: BMC Cardiovasc Disord. 2020 Nov 25;20:498. doi: 10.1186/s12872-020-01790-w (PMC7690024; doi:10.1186/s12872-020-01790-w)
Supplement: Supplementary file 1 — Additional file 1: Table S1. The urinary nitrate level of various cardiovascular diseases. [file 12872_2020_1790_MOESM1_ESM.doc]

Table S1. The urinary nitrate level of various cardiovascular diseases.

|  |  | Congestive Heart Failure | | Coronary Heart Disease | | Angina Pectoris | | Myocardial Infarction | |
| --- | --- | --- | --- | --- | --- | --- | --- | --- | --- |
|  |  | No | Yes | No | Yes | No | Yes | No | Yes |
| Urinary Nitrate (ng/mL) | All participants, n=14894 | 51935 (357) | 40137 (1608) | 51800 (359) | 46278 (1565) | 51764 (357) | 45096 (1762) | 51775 (359) | 47334 (1596) |
|  | Q1, n=3714 | 39866 (442) | 26565 (1434) | 39509 (436) | 32521 (2336) | 39463 (436) | 31037 (2248) | 39665 (441) | 29774 (1689) |
|  | Q2, n=3736 | 44290 (458) | 43006 (3452) | 44274 (461) | 43840 (2689) | 44271 (460) | 43613 (2979) | 44189 (459) | 46117 (2938) |
|  | Q3, n=3708 | 48043 (541) | 49604 (2662) | 47985 (541) | 51472 (3148) | 48096 (541) | 46603 (2547) | 47971 (543) | 51628 (2724) |
|  | Q4, n=3736 | 74919 (1054) | 69692 (6388) | 74968 (1063) | 69433 (3958) | 74937 (1057) | 69328 (5296) | 74887 (1064) | 73400 (4577) |

*Levels of urinary nitrate were divided into quartiles (Q1: ≤26100; Q2: 26100-45000; Q3: 45000-70600; Q4: >70600).
